# Hip Fracture Surgery in Trinidad and Tobago During the COVID-19 Pandemic: An Analysis of Two Patients Treated in the Parallel Healthcare System

**DOI:** 10.7759/cureus.30503

**Published:** 2022-10-20

**Authors:** Marlon Mencia, Reena Moonsie, Pedro Hernandez Cruz

**Affiliations:** 1 Surgery, The University of the West Indies, St. Augustine, TTO; 2 Department of Orthopaedics, Eric Williams Medical Sciences Complex, Champ Fleur, TTO; 3 Department of Surgery, Port of Spain General Hospital, Port of Spain, TTO

**Keywords:** covid-19 retro, trinidad & tobago, parallel health care system, dual-site, elderly, hip fracture

## Abstract

The rapid spread of the deadly coronavirus disease 2019 (COVID-19) pandemic has fundamentally affected healthcare delivery globally. As governments struggled to preserve life, several approaches to healthcare delivery have emerged. Central to limiting viral transmission is the separation of patients based on their COVID-19 status. Studies have shown that a geographically separate dual-site service is preferable, contingent upon the local infrastructure and circumstances. Despite the restrictions on free movement, most studies indicate that low-energy hip fractures in elderly patients have remained relatively constant throughout the pandemic. Arguably these patients represent the most vulnerable subgroup in society and are susceptible to developing severe COVID-19 respiratory disease.

In keeping with global recommendations, the government of Trinidad and Tobago devised a parallel healthcare system to limit the spread of disease. All regional health authorities under the Ministry of Health were at liberty to implement the system in a manner best suited for their particular infrastructure leading to highly variable practices among institutions. This report describes the clinical course of two hip fracture patients treated within the parallel healthcare system at different regional health authorities. Analysis of these cases provides an understanding of the potential risks to patients entering the parallel healthcare system and an insight into preventative measures to improve clinical outcomes.

## Introduction

Previous studies have projected that the number of hip fractures occurring in the world each year will rise to 6.26 million by 2050 [1,2]. To date, those predictions still remain valid. Moreover, despite the restrictions to free movement during the early stage of the pandemic, the incidence of hip fractures continues to be unaffected [3]. Elderly patients with hip fractures are especially vulnerable to developing severe coronavirus disease 2019 (COVID-19) illness, and hospital admission increases the risk of nosocomial infection [4,5]. Furthermore, there is compelling evidence that COVID-19-positive patients with hip fractures have higher 30-day mortality rates and increased morbidity compared to COVID-19-negative patients [6-9]. These findings required a concerted effort to reduce viral transmission and improve clinical outcomes. Early data from areas severely affected by the pandemic indicated that separate pathways for patients with and without the disease were the most critical element in providing a safe environment for patients and staff while maintaining hospital activity [10]. This was most effectively achieved by reorganising the surgical services on two sites with ‘COVID-19’ and ‘non-COVID-19’ hospitals using specialist teams [11-13].

In Trinidad and Tobago, a key containment strategy to reduce the spread of the virus was establishing a parallel healthcare system (PHCS), that is, a dual-site arrangement with specific hospitals designated for the treatment of confirmed COVID-19 cases separate from the routine public hospitals [14]. To accomplish that goal, the five regional health authorities (RHAs) that provide health services to the population reorganised the hospitals under their purview. This approach saw Trinidad and Tobago being ranked number one by the Oxford COVID-19 Government Response Tracker (OxCGRT) on May 1, 2020, in a report published by the University of Oxford [15].

As we reflect on the last two years with COVID-19, the overall success of the parallel healthcare system remains in question. For example, at the time of writing, there have been 4,195 deaths from COVID-19 in Trinidad and Tobago, the highest for any Caribbean territory [16]. The parallel healthcare system led to fundamental changes in service delivery, resource allocation and patient pathways, yet its effect on clinical outcomes remains largely undocumented. Elderly patients with hip fractures represent a significant trauma burden and were particularly vulnerable during the pandemic. One study from a hospital in south Trinidad found a delay in the surgical treatment of hip fractures during the pandemic but did not report morbidity and mortality data [17]. Furthermore, to date, there are no published studies on the outcomes of patients with hip fractures and COVID-19 infection undergoing surgical intervention in the parallel healthcare system. This study discusses the clinical journey of two hip fracture patients treated at different regional health authorities and examines the impact of the parallel healthcare system on their outcomes.

## Case presentation

Patient 1 treated at Regional Health Authority 1 

**Figure 1:**
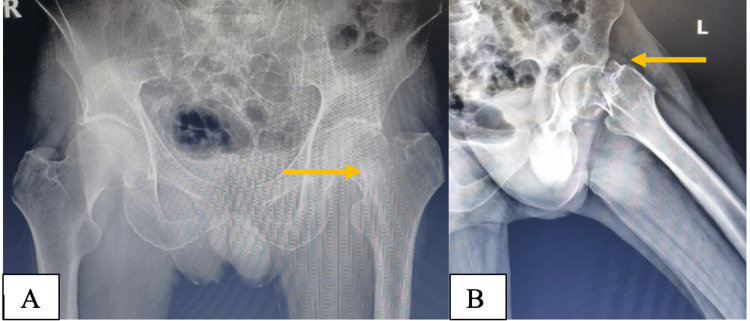
A- Anteroposterior pelvic radiograph. B- Lateral radiograph of the left hip. Both showing a displaced left hip fracture indicated by the yellow arrow.

A 63-year-old man attended the Emergency Department two weeks after falling at home. His medical history was unremarkable, but he was a heavy smoker (43 pack years). Radiographs showed a displaced intracapsular fracture of his left hip (Garden IV) (Figure 1).

**Figure 2:**
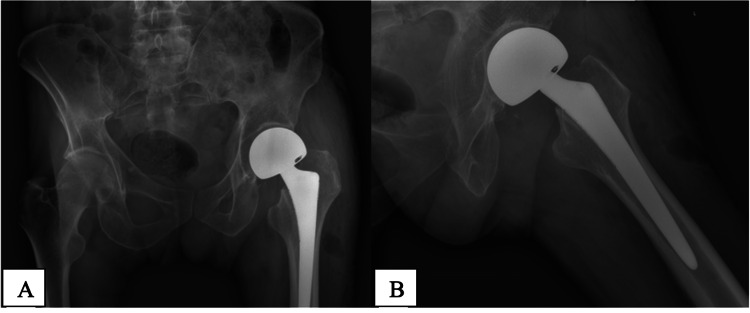
A- Anteroposterior pelvic radiograph. B- Lateral radiograph of the left hip. Both showing a well-positioned left bipolar hemiarthroplasty.

Under the hospital protocol, he was kept in a holding area within the Emergency Department while awaiting the results of his COVID-19 polymerase chain reaction (PCR) test. After receiving a negative result, he was admitted to a four-bed bay on a COVID-free ward and advised to wear a facemask. His surgery was delayed for two weeks and although he was asymptomatic, routine retesting within 48 hours of surgery returned a positive COVID-19 PCR test. The patient underwent a left hemiarthroplasty in a theatre reserved for COVID-19-positive patients (Figure 2).

Postoperatively, he remained afebrile without respiratory symptoms and was transferred to the PHCS for rehabilitation.

The patient seemed to recover well and was ambulating comfortably with a walker; however, on day nine began experiencing breathlessness. His oxygen saturations were low on room air (93-95%) and he was tachycardic. Treatment was started with oxygen supplementation, dexamethasone and intravenous antibiotics.

**Figure 3:**
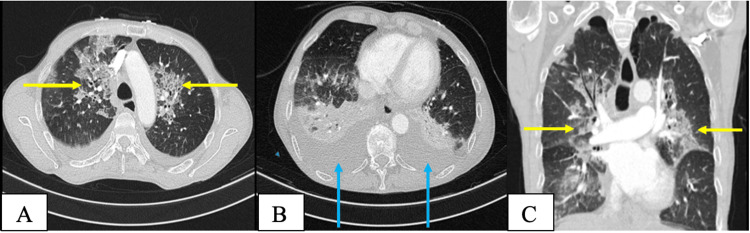
Computed tomography of the chest. A & B-Axial views, C- Coronal view. Yellow arrows indicate areas of atelectasis, blue arrows indicate bilateral pleural effusion.

His chest radiograph demonstrated bilateral infiltrates throughout the lung fields, suggestive of acute respiratory distress syndrome (ARDS). A computed tomography pulmonary angiogram (CTPA) ruled out a pulmonary embolism and showed bilateral pleural effusions, pulmonary emphysema and atelectasis (Chest CT severity score 19/25: severe disease) (Figure 3) [18].

In light of these results, he was admitted to the Intensive Care Unit and placed on continuous passive air pressure (CPAP) using tiotropium bromide and salbutamol. Despite these measures, he continued to deteriorate and was prophylactically intubated on day 19. He returned a negative swab on day 25 but could not be taken off ventilatory support and eventually succumbed to COVID-19-related pulmonary complications 42 days after surgery.

Patient 2 treated at Regional Health Authority 2 

A 61-year-old fully vaccinated man presented to the Emergency Department with a displaced intertrochanteric fracture of his right hip. His past medical history was unremarkable, and he neither smoked cigarettes nor consumed alcohol. Although asymptomatic, his pre-admission COVID-19 PCR was positive, and the patient was transferred to the PHCS. While in the PHCS, he remained asymptomatic and was transferred back to the general hospital 28 days after a negative COVID-19 PCR test.

**Figure 4:**
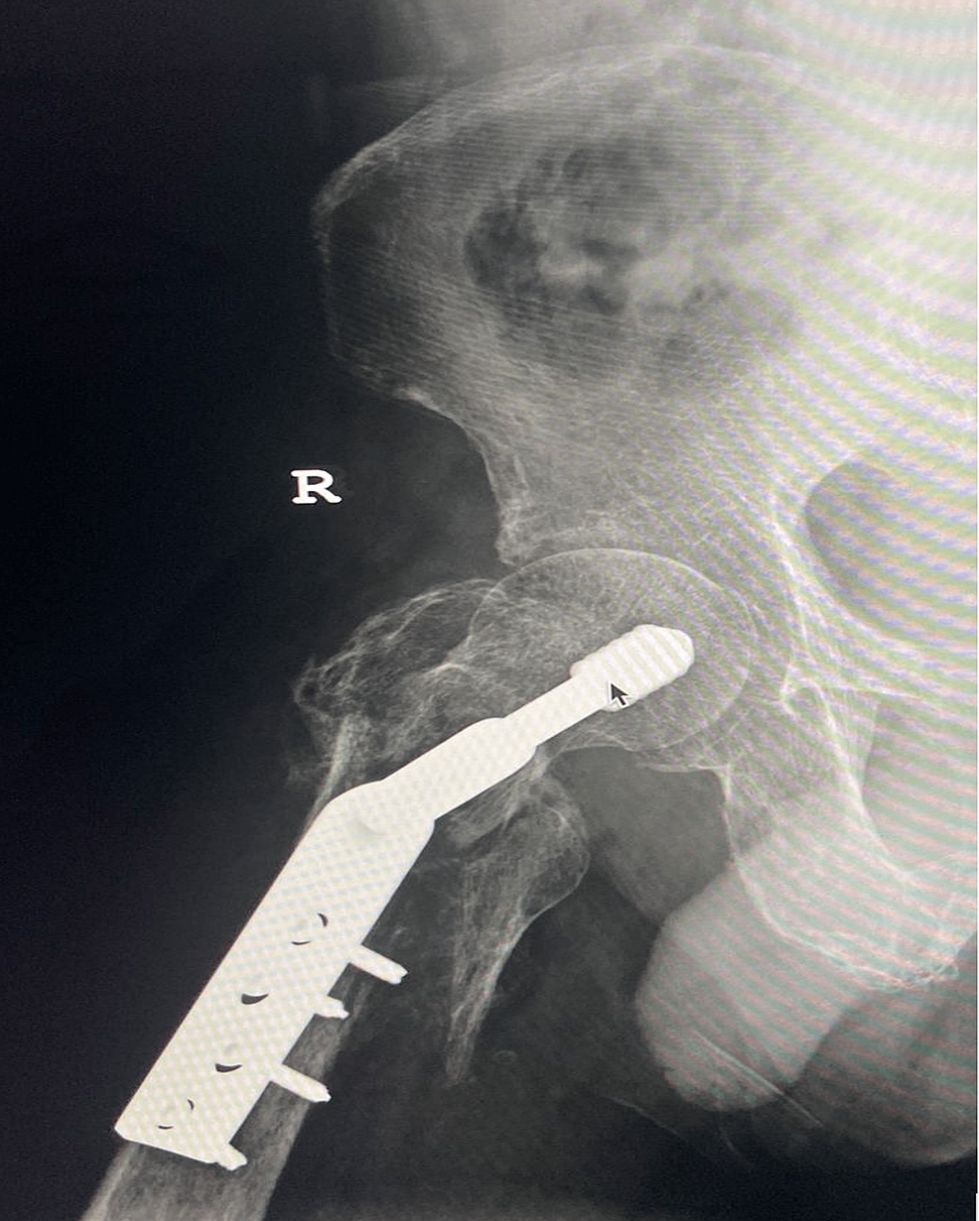
Anteroposterior radiograph of the right hip in extreme external rotation showing an unacceptable fracture reduction.

A dynamic hip screw (DHS) was used to stabilise the fracture, and the patient recovered well. He was discharged home, where his rehabilitation was hampered by persistent severe hip pain and inability to walk. His fear of contracting COVID-19 resulted in a delay in seeking further medical attention; however, with no progress being made, he eventually returned to the hospital. At this presentation, his right lower limb was significantly shortened and externally rotated. Radiographs showed that the fracture had not been reduced satisfactorily, but there was no evidence of implant failure (Figure 4).

**Figure 5:**
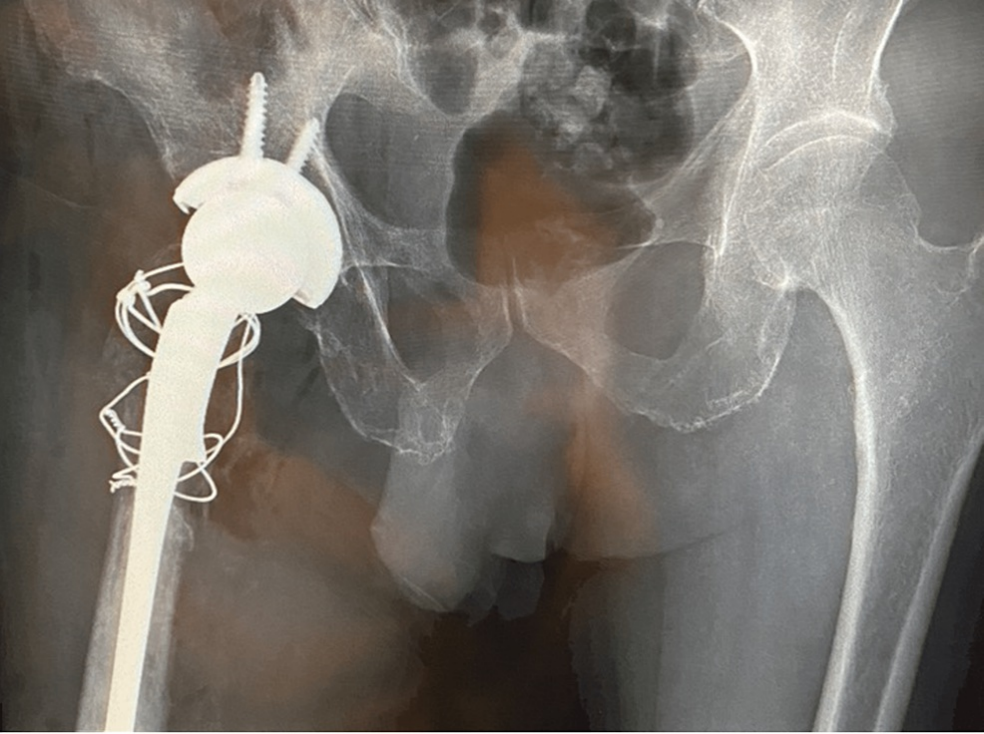
Anteroposterior radiograph of the pelvis showing the reconstructed right total hip arthroplasty.

We agreed that revising the fixation to a total hip arthroplasty would be the best option to promote rapid mobilisation. At surgery, we encountered extensive fibrosis and marked chondrolysis within the hip joint with little hard callus. The hip was reconstructed using a porous-coated shell and a cemented calcar-replacement stem, while the greater trochanter and posteromedial calcar were reattached with cerclage wires (Figure 5). Tissue samples showed no growth after 48 hours. 

**Figure 6:**
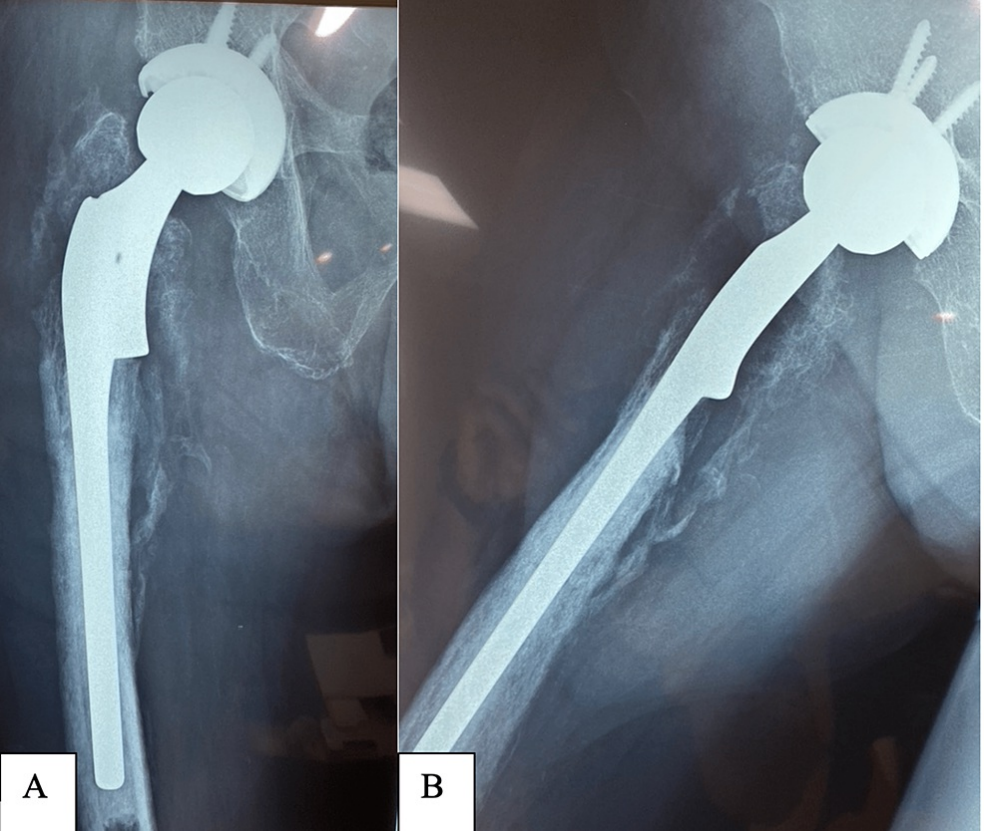
Anteroposterior (A) and lateral radiographs (B) of the total hip arthroplasty showing outline ossification of the proximal femur with formation of a neo-cortex.

After surgery, the patient reported significantly less pain and could mobilise comfortably with a walker. One week later, we noted a persistent wound discharge and took the patient back to the theatre. At surgery, we found marked osteonecrosis of the posteromedial calcar fragment and parts of the greater trochanter with loosening of the cerclage wires. All wires were removed, the soft tissues debrided, and the wound irrigated with copious dilute betadine solution. Despite losing the abductor muscle attachment, the hip remained stable, and the wound was closed over a suction drain. Culture reports were again negative, and the patient was discharged after seven days of intravenous antibiotics and placed on a three-month course of oral antibiotics. Six weeks after surgery, the incision is fully healed, the patient remains pain-free and has resumed rehabilitation (Figure 6).

## Discussion

Several reports have documented inferior clinical results in hip fracture patients during the pandemic compared with the pre-pandemic era [6-9]. Where adequate infrastructural capacity exists, dual-site services are recommended to improve patient care. However, a more granular appreciation of logistical and clinical issues is critical to avoid errors that may result in suboptimal outcomes [11].

Nosocomial transmission

Using one hospital to treat both COVID-19-positive and -negative patients increases the risk of cross-contamination. Despite efforts to limit contagion, transmission to hospitalised patients is common and nosocomial transmission is recognised as an important route of spread [19-21]. 

Strict precautions within the hospital were enforced to reduce transmission, including limiting visitors, social distancing and frequent hand sanitisation. Nevertheless, patients remain vulnerable to contracting COVID-19 while awaiting surgery, as was the case with Patient 1. Additional protection could have been provided by, first, prioritising surgical stabilisation to minimise the time in the hospital, thereby reducing exposure. Second, divide the orthopaedic service into independent teams of two orthopaedic units. Teams treat either COVID-19-positive or -negative patients, and units rotate to allow adequate rest before returning to work [22]. Third, restricted orthopaedic wards to effectively “ring-fence” beds and expedite surgery. Hip fracture patients should be protected from COVID-19 infection. Specialised staff using separate wards facilitates quality care, reduces hospital stay, and lowers the risk of contracting COVID-19 in hospitals [23,24].

Hip surgery in COVID-19-positive patients 

It is widely accepted that early surgery produces better outcomes in hip fracture patients [25]. However, when combined with COVID-19 infection, hip fracture surgery is associated with significantly higher morbidity and 30-day mortality rates. A multicentre retrospective study of six trauma centres by the IMPACT-Scot Study Group reported a 30-day mortality of 35.5% in COVID-19-positive patients following an acute hip fracture [21]. Tripathy et al., in a systematic review and meta-analysis, reported similar findings of increased 30-day mortality (32.23% COVID-19 positive vs 8.85% COVID-19 negative) [8]. Despite these poor results, early surgery is recommended for asymptomatic patients [26]. It is important to note that Patient 2 remained asymptomatic throughout his hospital stay. The hospital protocols at that time dictated that all COVID-19-positive patients who did not require urgent surgery should be transferred to the PHCS to await a negative swab before returning to the main hospital. To the best of our knowledge, this protocol was not used in other countries and is without scientific merit. It appears that this was done primarily to permit the main hospital to operate as a COVID-19-free site and maintain routine services. Administratively, hip fractures were classified as non-urgent, and surgery was not prioritised, resulting in unanticipated complications, as illustrated by this case. While the treatment of COVID-19-positive patients must be adapted to the local resources, this complication may have been avoided if the following points were observed: more reliance should have been placed on the scientific literature; greater collaboration with all clinicians was required, in this case, the orthopaedic surgeons; and establishment of trauma services within the PHCS.

Dual-site service 

During the pandemic, better clinical outcomes were seen in patients treated in a dual-site system compared with the traditional single-site model [11,27]. A dual-site system aims to deliver equal treatment at geographically separate sites to patients based on their COVID-19 infection status. One of the most beneficial aspects of this arrangement is a reduction in nosocomial transmission. A prospective comparison of single vs dual-site service reconfiguration for hip fractures during the pandemic found greater nosocomial transmission for single-site services [11]. Another study found time to surgery to be longer at the ‘COVID’ sites compared with ‘COVID-free’ sites with no increase in mortality [27]. Overall, in a scoping review examining dual vs single-site services during the pandemic, a dual-site configuration was generally favoured where the hospital infrastructure allowed such an arrangement [11]. 

Both RHAs implemented a dual-site service model but failed to duplicate orthopaedic services at each site, which contributed to the unfortunate outcomes for these two patients. Equality of service is critical for a dual-site arrangement but presents particular logistical challenges; orthopaedic staff would have to be divided between the two sites and the existing operating theatres opened. Despite the local limitations, successful dual-site services have been reported by Ramsingh et al. in a paper on urgent and emergency cardiac surgery in Trinidad [28].

## Conclusions

Trinidad and Tobago’s parallel healthcare system, a type of dual-site service, was initially successful at containing and limiting the spread of COVID-19 infection. However, an effective dual-site system separates patients based on their infection status without delaying treatment of concurrent illness. That arrangement requires the duplication of services at each site, which was not a feature of the parallel healthcare system and ultimately contributed to inferior clinical outcomes. With the end of the pandemic in sight, policymakers must use the lessons learnt over the last two years to restructure the health services to protect the most vulnerable in society from future pandemics.
